# Antitumor Activity of Sodium Selenite, Palbociclib, and Disulfiram Against Osteosarcoma and Rhabdomyosarcoma Cell Lines

**DOI:** 10.1002/ddr.70304

**Published:** 2026-05-03

**Authors:** María Ángeles Chico, Kevin Doello, Raul Ortiz, Mercedes Peña, Consolación Melguizo, Cristina Mesas, Jose Prados

**Affiliations:** ^1^ Institute of Biopathology and Regenerative Medicine (IBIMER), Center of Biomedical Research (CIBM) Granada Spain; ^2^ Instituto de Investigación Biosanitaria de Granada, ibs.GRANADA University of Granada Granada Spain; ^3^ Department of Anatomy and Embryology, Faculty of Medicine University of Granada Granada Spain; ^4^ Medical Oncology Service, Virgen de las Nieves Hospital Granada Spain

**Keywords:** disulfiram, osteosarcoma, palbociclib, rhabdomyosarcoma, sodium selenite

## Abstract

Osteosarcoma and rhabdomyosarcoma are the most common pediatric sarcomas, yet prognosis remains poor due to high relapse rates. This study investigates the repurposing of palbociclib (PB) and disulfiram (DS), alongside sodium selenite (SS), as potential therapeutic strategies. Using cell lines, we assessed antiproliferative effects via Sulforhodamine B, colony formation, and wound healing assays. Mechanisms of action were explored through protein expression of PARP‐1 (apoptosis) and LC3β (autophagy), qPCR for stem cell markers, and ROS quantification. Finally, antitumor and anti‐angiogenic efficacy was validated using the in ovo chicken chorioallantoic membrane (CAM) assay. Results demonstrated that all three compounds inhibited proliferation, migration, and spheroid growth while inducing apoptosis and autophagy. Notably, SS and PB elevated ROS levels, triggering parthanatos‐mediated cell death via AIF nuclear translocation. SS also exhibited significant anti‐angiogenic activity. Xenograft CAM models confirmed the in vivo efficacy of SS, PB, and DS against RD and MG63 cells. These findings suggest that SS, PB, and DS are promising candidates for pediatric sarcoma treatment, particularly as maintenance therapies to prevent relapse following conventional radical treatment.

## 1 Introduction

Sarcomas are rare malignant tumors of mesenchymal origin, representing about 1% of adult cancers but 20% in children and adolescents, making them primarily pediatric (Grünewald et al. 2020). They are divided into bone sarcomas (20%), mainly osteosarcoma, and soft tissue sarcomas (STS) (80%), where rhabdomyosarcoma is most common in children (Beird et al. 2022; Weiss and Harrison 2024). Currently, standard therapy combines surgery and chemotherapy, but drug resistance and metastasis lead to poor outcomes (Kelley et al. 2020). The 5‐year survival rate is ~60% for localized and 30% for metastatic osteosarcoma (Roberts et al. 2019), and 74% versus 29% for embryonal and alveolar rhabdomyosarcoma, respectively (Amer et al. 2019). Despite initial chemosensitivity, relapse is frequent, highlighting the urgent need for new therapies (Al Shihabi et al. 2024).

Drug repurposing identifies new cancer applications for existing drugs, enabling faster development through known safety profiles (Aggarwal et al. 2021; Fu et al. 2022). Palbociclib (PB), a CDK4/6 inhibitor (Finn et al. 2015), was FDA‐approved in 2015 with letrozole for ER + /HER2‐ metastatic breast cancer (Xu et al. 2022) and later confirmed effective with fulvestrant (Turner et al. 2018). Moreover, PB also shows activity in lung cancer (Gao et al. 2024) and CDK‐overexpressing sarcoma (Kohlmeyer et al. 2020; Martin‐Broto et al. 2023). Disulfiram (DS), an ALDH inhibitor for alcoholism, and sodium selenite (SS), a selenium compound, both induce ROS (reactive oxygen species) mediated apoptosis, showing promising antitumor potential (Guo et al. 2021; Xu et al. 2023; Yang et al. 2018).

The aim of this study was to evaluate the antitumor activity of PB, SS, and DS in osteosarcoma and rhabdomyosarcoma cell lines and to investigate the molecular mechanisms underlying their effects. Ultimately, this research sought to identify effective drugs against these tumors, with the potential to be used as maintenance therapy after radical treatment.

## 2 Materials and Methods

### 2.1 Drugs

SS, PB and DS were obtained from Sigma‐Aldrich (Madrid, Spain). DS was dissolved in dimethyl sulfoxide (DMSO) (Sigma‐Aldrich, Madrid, Spain), whereas SS and PB were dissolved in Dulbecco's Modified Eagle Medium (DMEM) (Sigma‐Aldrich, Madrid, Spain). Doxorubicin (DX), also obtained from Sigma‐Aldrich (Madrid, Spain), was used as a chemotherapeutic agent control.

### 2.2 Cell Culture

Osteosarcoma (MG63 and Saos‐2) and rhabdomyosarcoma (RD) cell lines were obtained from the Scientific Instrumentation Centre (CIC) of the University of Granada. MG63 and RD were cultured in DMEM (Sigma‐Aldrich, Madrid, Spain). Saos‐2 cells were maintained in McCoy's 5A medium (Sigma‐Aldrich, Madrid, Spain). Culture media were supplemented with 10% fetal bovine serum (FBS) (ThermoFisher, Waltham, MA, USA) and 1% penicillin/streptomycin (Sigma‐Aldrich, Madrid, Spain). Cultures were incubated at 37°C in a humidified atmosphere with 5% CO_2_.

### 2.3 Cell Proliferation Assay

Cells were seeded in 48‐well plates at a density of 1.5 × 10^4^ cells/well for RD, 8 × 10^3^ cells/well for MG63, and 1 × 10^4^ cells/well for Saos‐2. After 24 h, cells were treated with increasing concentrations of the drugs for 72 h and stained with sulforhodamine B (SRB) (Ortigosa‐Palomo et al. 2023). Optical density (OD) was measured at 492 nm using the 800™ TS plate reader (BioTek, Madrid, Spain). Cell proliferation (%) was calculated using the following formula (1):

$$\mathrm{Proliferation}\;\left(\%\right)=\frac{\mathrm{OD}\;\mathrm{sample}-\mathrm{OD}\;\mathrm{blank}}{\mathrm{OD}\;\mathrm{negative}\;\mathrm{control}-\mathrm{OD}\;\mathrm{blank}}\;\times \;100$$

### 2.4 Wound Healing Assay

To analyze cell migration capacity, RD and Saos‐2 cells (3 × 10⁵/well) and MG63 cells (1.5 × 10⁵/well) were seeded in 12‐well plates. After 24 h, a vertical scratch was made with a pipette tip, and the medium was replaced with serum‐free DMEM or McCoy's 5 A. Cells were then treated with IC₅ doses of the respective drugs. Images were taken at various time points, and migration was quantified as the reduction of the cell‐free area using the Chemotaxis and Migration Tool in ImageJ software.

### 2.5 Clonogenic Assay

Cell lines were treated with IC_50_ doses for 72 h and viable cells were subsequently seeded in 12‐well plates at a density of 200 cells per well. MG63, RD, and Saos‐2 colonies were grown for 10, 14, and 18 days, respectively, fixed with TCA (Sigma‐Aldrich, Madrid, Spain) for 20 min at 4°C and stained with 0.08% SRB (Sigma‐Aldrich, Madrid, Spain) in 1% acetic acid (PanReac AppliChem, Barcelona, Spain) for 20 min at room temperature. Images were captured, and the number of colonies was quantified using ImageJ software.

### 2.6 Multicellular Tumor Spheroid Formation Assays

Multicellular tumor spheroids (MTS) were generated from sarcoma cell lines. MG63 and RD cells (1.5 × 10⁴/well) and Saos‐2 cells (3 × 10⁴/well) were seeded in 96‐well plates coated with 50 µL of 1% (w/v) agarose. After centrifugation (2200 rpm, 10 min) and incubation (37°C, 5% CO₂, 72 h), formed MTS were treated with 5 × IC₅₀ doses for two 72 h cycles. Vehicle‐treated spheroids served as negative controls. Spheroid dimensions (longest diameter (LD) and shortest diameter (SD)) were measured from microscope images (Olympus, CKX41) using ImageJ software. The MTS volume was calculated using the formula (2):

$$\mathrm{Volume}=\left(\mathrm{LD}\;x\;{\mathrm{SD}}^{2}\right)\times \frac{\pi }{6}$$

In addition, MTS viability was assessed using the Cell Counting Kit‐8 (CCK‐8) kit from Dojindo Laboratories (Kumamoto, Japan). Spheroids medium was replaced with medium containing 10% CCK‐8, and incubated at 37°C for 3 h. After which absorbance at 450 nm was measured using the 800™ TS plate reader (BioTek, Madrid, Spain). The percentage of proliferation was calculated using the previously described formula (1).

### 2.7 Western Blot Assay

RD, MG63 and Saos‐2 cells (3 × 10^5^) were treated with 2xIC_50_ concentrations of DX, SS, PB and DS for 24 h. Total proteins were extracted using RIPA buffer (G‐Biosciences, Saint Louis, USA). Protein concentration was quantified using Bradford's reagent (Sigma‐Aldrich, Madrid, Spain). A total of 45 μg of proteins were denatured, separated by electrophoresis, and transferred to nitrocellulose membranes protocol (Ortigosa‐Palomo et al. 2023). Membranes were incubated overnight at 4°C with primary antibodies: rabbit anti‐VEGFA antibody (1:1000, ab46154, Abcam), rabbit anti‐PARP1 antibody (1:1000, ab32138, Abcam) and rabbit anti‐LC3β antibody (1:1000, NB100‐2220, Novus Biologicals). Then, they were incubated for 1 h at room temperature with anti‐mouse IgGκ BP‐HRP secondary antibody (1:5000, sc‐516102, Santa Cruz Biotechnology) or anti‐rabbit IgG‐HRP secondary antibody (1:5000, sc‐2357, Santa Cruz Biotechnology). β‐actin expression (1:25000, A3854, Sigma‐Aldrich) was used as internal control. Finally, proteins were developed with Amersham™ ECL™ Prime (Thermo Fisher Scientific, Massachusetts, USA) on LAS‐4000 mini (GE Healthcare). The bands obtained were quantified using Quantity One 4.6.8 software (Bio‐Rad, California, USA).

### 2.8 N‐Acetyl‐L‐Cysteine Combination Proliferation Assay

The role of ROS in drug‐induced cytotoxicity was assessed using the antioxidant agent N‐acetyl‐L‐cysteine (NAC) (Sigma‐Aldrich, Madrid, Spain). RD, MG63, and Saos‐2 cell lines were seeded in 48‐well plates at densities of 1.5 × 10⁴, 8 × 10³, and 1 × 10⁴ cells/well, respectively. After 24 h, cells were pre‐treated with a non‐cytotoxic dose of NAC (2 mM) for 2 h. Subsequently, they were treated with IC₅₀ doses of DX, SS, PB, and DS for 72 h. SRB staining was performed as previously described.

### 2.9 RNA Isolation and RT‐qPCR

The cell lines RD, MG63 and Saos‐2 were seeded at 3 × 10^5^ cells in T25 flasks. After 24 h, cells were treated with IC_50_ doses of DX, SS, PB and DS for 72 h. Cells were trypsinized, centrifuged and resuspended in Trizol Reagent (Sigma‐Aldrich, Madrid, Spain). RNA was extracted using the RNeasy Mini Kit (Qiagen, Hilden, Germany), quantified with Nanodrop 2000 (Thermo Fisher, Massachusetts, USA), and reverse‐transcribed into cDNA with Reverse Transcription System kit (Promega, Wisconsin, USA). RT‐qPCR was performed with TB Green Premix Ex Taq II (Takara Bio, Kusatsu, Japan) following the manufacturer's instructions and StepOnePlus™ Real‐Time PCR System (Thermo Fisher, Massachusetts, USA). The primers used and sequences are shown in Table S1. Finally, gene expression was calculated using the 2‐∆∆Ct formula.

### 2.10 Chick Chorioallantoic Membrane Assay

According to EU legislation, chick embryo chorioallantoic membrane (CAM) trials do not require Ethics Committee approval (Mitrevska et al. 2023). Fertilized chicken (*Gallus domesticus*) eggs were obtained from the certified farm Santa Isabel (Cordoba, Andalusia, Spain) and incubated horizontally at 37°C and 60% humidity. On embryonic day 3 (ED3) a small hole was opened. On ED7, five groups (*n* = 10) were established, and a sterile plastic ring was placed on the CAM, where 1 × 10^6^ RD or MG63 cells per egg were directly pipetted in 15 μL of Matrigel (Thermo Fisher Scientific). The success of xenograft development was visually confirmed on ED9. On this day, tumors were treated with 40 µL of a 5xIC_50_ concentration of DX, SS, PB, and DS. After 24 h, the ring was removed. Finally, on ED13, tumor volume was calculated using the following formula (3):

$$\mathrm{Volume}\;\left({\mathrm{mm}}^{3}\right)=\left(a\times b^{2}\right)\times \frac{\pi }{6}$$

Where “a” is the longest diameter and “b” is perpendicular diameter.

Chick embryos were euthanized by decapitation, and tumors were resected from the CAM, fixed in 4% paraformaldehyde (PanReac, Barcelona, Spain) for 24 h and embedded in paraffin to obtain 5 µm sections using a rotary microtome (Leica, Wetzlar, Germany). After deparaffinization and hydration, sections were stained with hematoxylin‐eosin (H&E) and pentachrome stain (Doello 2014). Images were captured using a Leica microscope (Leica DM IL LED, Leica Microsystems, Wetzlar, Germany).

### 2.11 Inmunofluorescence Analysis

Tumor sections were deparaffinized, hydrated and subjected to antigen retrieval with 20% citrate buffer for 20 min in a heat steamer. Sections were blocked with 0.5% goat serum solution in 0.1% PBS‐Tween 20 and 0.3% Triton X‐100 for 1 h at room temperature and incubated for 1 h with primary antibodies: Desmin (1:500, ab15200, Abcam), Osteopontin (1:250, ab8448, Abcam), CD45 (1:100, 345809, Becton Dickinson) and Ki‐67 (1:50, 550906, BD BioSciences). Subsequently, Alexa‐647® secondary antibody (1:200, Cell Signaling Technologies) was incubated for 30 min. Nuclei were stained with Hoechst (1:1000) for 10 min. Images were captured by fluorescence microscope (Leica DM IL LED, Leica Microsystems, Wetzlar, Germany).

### 2.12 DNA Fragmentation Assay (TUNEL)

Sections were deparaffinized, hydrated, and permeabilized with a 0.1% sodium citrate and 0.1% Triton X‐100 in PBS for 8 min at room temperature. After PBS washing, the TUNEL protocol was carried out following the manufacturer's instructions of the TMR red in situ cell death detection kit (Roche, Basel, Switzerland). Nuclei were stained with Hoechst (1:1000) for 10 min. Finally, images were captured using fluorescence microscopy (Leica).

### 2.13 Hen's Egg Test for Micronucleus Induction

Fertilized eggs were incubated at 37°C and 60% humidity, blunt end up. On ED8, a small hole was made and 60 µL of a 5IC_50_ concentration of DX, SS, PB, and DS was applied. Cyclophosphamide (CP) (MedChem, Sollentuna, Sweden) at 50 µg/egg was the positive control. After 72 h, 8 µL of blood was collected, fixed with 100% methanol for 10 min, and stained with Hoechst (1:1000) for 5 min. Micronuclei were visualized under a fluorescence microscope (Leica), analyzing 1000 cells to determine frequency.

### 2.14 Statistical Analyses

Statistical analyses were performed with GraphPad Prism 9 software. Results were represented as mean ± standard deviation (SD). One‐way ANOVA (univariate comparison) and two‐way ANOVA (comparison of two variables) with Tukey's post‐hoc test was performed to analyze comparisons between two samples. Statistical significance was considered when a *p*‐value < 0.05 was obtained.

## 3 Results

### 3.1 Antiproliferative Activity of Treatments in Sarcoma Cell Lines

The cytotoxicity of SS, PB, and DS was evaluated in osteosarcoma (MG63, Saos‐2) and rhabdomyosarcoma (RD) cell lines after 72 h of treatment, with DX included as a reference drug. As shown in Table 1, DS exhibited the strongest antiproliferative effect on RD and Saos‐2 cell lines, with IC_50_ values (0.19 and 0.22 µM, respectively) similar to those observed with DX. For all IC_50_ determinations, DMSO was non‐toxic and was therefore used as a control in the assays. Only in MG63 cells the IC_50_ of DS increased to 25.97 µM. SS exhibited low IC_50_ values in the three tested cell lines, RD, MG63, and Saos‐2 (6.03, 8.76, and 7 µM, respectively), while PB showed the lowest activity against all cell lines with IC_50_ values ranging from 13.25 to 69.77 µM.

**TABLE 1:** IC_50_ values (µM) of the different drugs in sarcoma cell lines.

	IC_50_ (µM)		
Drugs	RD	MG63	Saos‐2
Selenite	6.03 ± 0.08	8.76 ± 1.47	7.00 ± 0.41
Palbociclib	13.25 ± 2.74	69.77 ± 5.57	33.37 ± 2.53
Disulfiram	0.19 ± 0.02	25.97 ± 1.81	0.22 ± 0.01
Doxorubicin	0.15 ± 0.02	0.16 ± 0.02	0.97 0.01

### 3.2 Effect of Treatments on Tumor Sarcoma Aggressiveness

**FIGURE 1:**
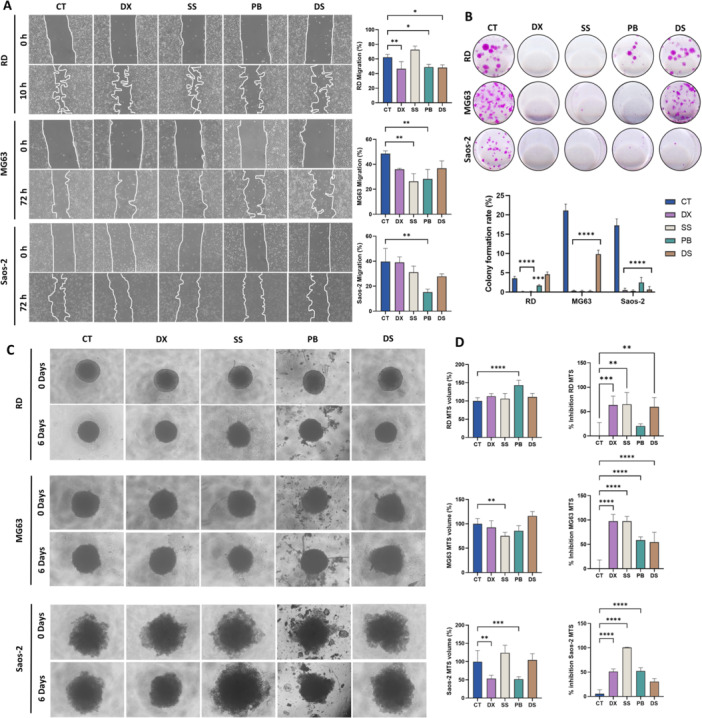
Effect of treatments on tumor multicellular spheroids and aggressiveness in sarcoma cell lines. (A) Effect of DX, SS, PB and DS on cell migration (Images were acquired at 4X magnification). (B) Colony‐forming ability in RD, MG63 and Saos‐2 cell lines. (C) Representative microscopy images of RD, MG63 and Saos‐2 MTS treated with 5IC_50_ of DX, SS, PB and DS on day 0 and day 6 (Images were acquired at 4X magnification). (D) Graphical representation of MTS volume modulation and inhibition of cell viability assessed by CCK8 assays after treatment. MTS treated with vehicle were used as controls. Data are presented as mean ± standard deviation. Statistical significance relative to the control was determined as follows: *p < 0.05, **p < 0.01, ***p < 0.001 and ****p < 0.0001.

Cell migration assay during 72 h was carried out (Figure 1A). Only a 10 h drug exposure was used in RD due to the high migration rate of this cell line. PB significantly reduced migration capacity in the three cell lines, RD, MG63, and Saos‐2 (13.4%, 20.3%, and 24.4%, respectively) compared to the control. By contrast, DS and SS only significantly reduced migration in RD (13.9%) and MG63 (22.2%) cells, respectively. On the other hand, a colony‐forming capacity assay was used to determine tumor aggressiveness. As shown in Figure 1B, sarcoma cell lines pre‐treated for 72 h with IC₅₀ concentrations of SS or PB showed an almost complete loss of colony‐forming ability. DS produced a comparable effect in Saos‐2 cells but not in RD or MG63.

### 3.3 Effect of Treatments on Multicellular Tumor Spheroid From Sarcoma Cell Lines

MTS were generated from RD, MG63, and Saos‐2 sarcoma cell lines to assess the antitumor effects of SS, PB, and DS in three‐dimensional in vitro tumor models, which resemble in vivo model conditions. As shown in Figure 1C, SS was highly effective, significantly reducing cell viability in RD, MG63 and Saos‐2 MTS (65%, 97.5% and 100%, respectively) compared to the control. However, SS only significantly reduced the volume of MG63 MTS by 24.7%. In addition, PB treatment significantly inhibited viability in MG63 (58.8%) and Saos‐2 MTS (52.5%), while DS inhibited RD MTS by 60% and MG63 MTS by 54.6%. However, no significant reduction in MTS volume was observed following PB or DS treatment, except for PB in Saos‐2 (Figure 1D).

### 3.4 Determination of Molecular Mechanisms Induced by Treatments

**FIGURE 2:**
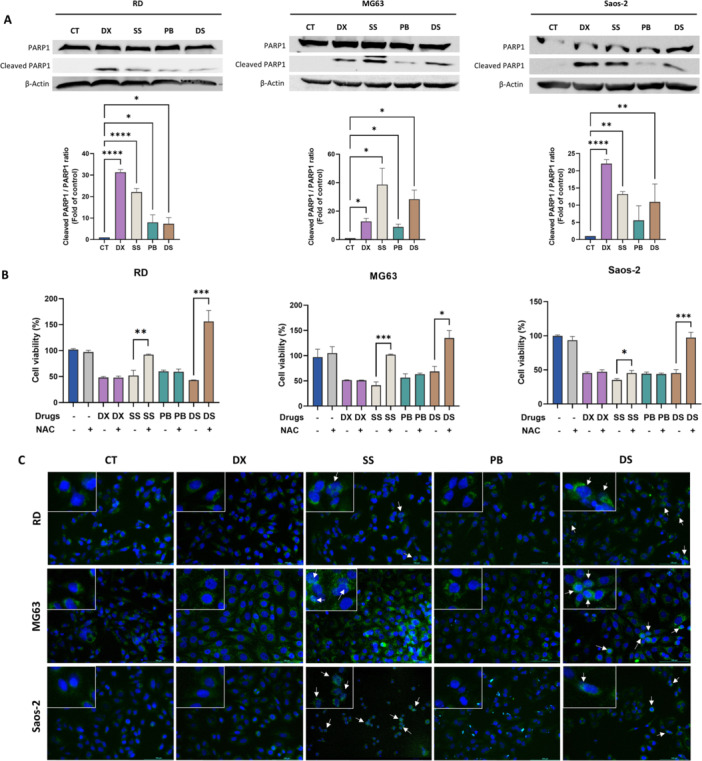
Induction of apoptosis by treatments in sarcoma cell lines. (A) Western blot analysis of PARP1 protein significance relative to the control was determined as follows: *p < 0.05, **p < 0.01, ***p < 0.001, expression in cell lines treated with DX, SS, PB, and DS. β‐actin was used as an internal control. (B) Graphical representation of cell viability modulation with and without NAC pre‐treatment in RD, MG63, and Saos‐2 cell lines treated with DX, SS, PB, and DS. (C) Immunofluorescence images of AIF expression in RD, MG63, and Saos‐2 cells treated with DX, SS, PB, and DS. Images were acquired at 20X magnification. Statistical and ****p < 0.0001.

**FIGURE 3:**
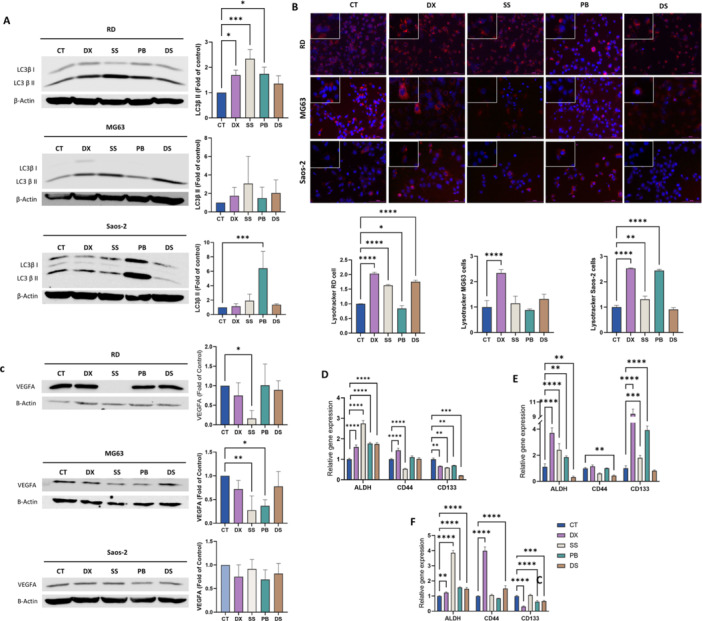
Effect of treatments on induction of autophagy, angiogenesis and CSC expression in sarcoma cell lines. (A) Western blot analysis of LC3β protein expression in cell lines treated with DX, SS, PB, and DS. β‐actin was used as an internal control. (B) Fluorescence microscopy images and graphical representation of the LysoTracker assay after treatment with DX, SS, PB, and DS in RD, MG63, and Saos‐2 cell lines. Images were acquired at 20X magnification. (C) Western blot analysis of VEGFA protein expression in cell lines treated with DX, SS, PB, and DS. β‐actin was used as an internal control. Graphical representation of the relative expression levels obtained by RT‐qPCR for the CSC markers ALDH, CD44, and CD133 after treatment with DX, SS, PB, and DS (72 h) in the sarcoma cell lines (D) RD, (E) MG63, and (F) Saos‐2. Statistical significance relative to the control was determined as follows: *p < 0.05, **p < 0.01, ***p < 0.001, and ****p < 0.0001.

Western blot analysis showed cleaved PARP1 overexpression after treatment with DX, SS, PB and DS for 24 h in all cell lines, except for PB in Saos‐2 cells (Figure 2, 3). We also examined NAC co‐treatment. NAC pretreatment followed by SS and DS increased cell viability, with no changes under other conditions (Figure 2, 3). Immunofluorescence showed SS and DS induced strong AIF nuclear translocation, unlike DX and PB (Figure 2, 3).

The expression of LC3β, a key protein in the autophagy process, was analyzed. Western blot analysis revealed an increase in LC3β II expression after treatment with DX and SS in the RD cell line, as well as with PB in both RD and Saos‐2 cell lines, indicating autophagy‐mediated cell death. However, no significant increase in LC3β II expression was observed in MG63 cells (Figure 2, 3). Lysosomal vesicle formation, assessed by Lysotracker staining increased in RD cells with all treatments and in Saos‐2 cells, except DS (Figure 2, 3). However, in MG63 cells, only DX increased the Lysotracker signal.

Finally, VEGFA analysis showed that SS significantly reduced its expression in RD and MG63 cell lines, while PB reduced VEGFA expression in MG63 cells. No significant differences were observed in the Saos‐2 cell line (Figure 2, 3).

### 3.5 Modulation of Cancer Stem Cell Markers

RT‐qPCR was employed to study the modulation of cancer stem cell (CSC) marker expression after DX, SS, PB, and DS treatment. Our results showed that all treatments reduced CD133 expression in RD cell line. In addition, SS reduced CD44 as well (Figure 2, 3). DS reduced the expression of ALDH and CD44 in the MG63 cell line (Figure 2, 3). Finally, PB and DS reduced CD133 expression in Saos‐2 (Figure 2, 3).

### 3.6 In Ovo Antitumor Assay

**FIGURE 4:**
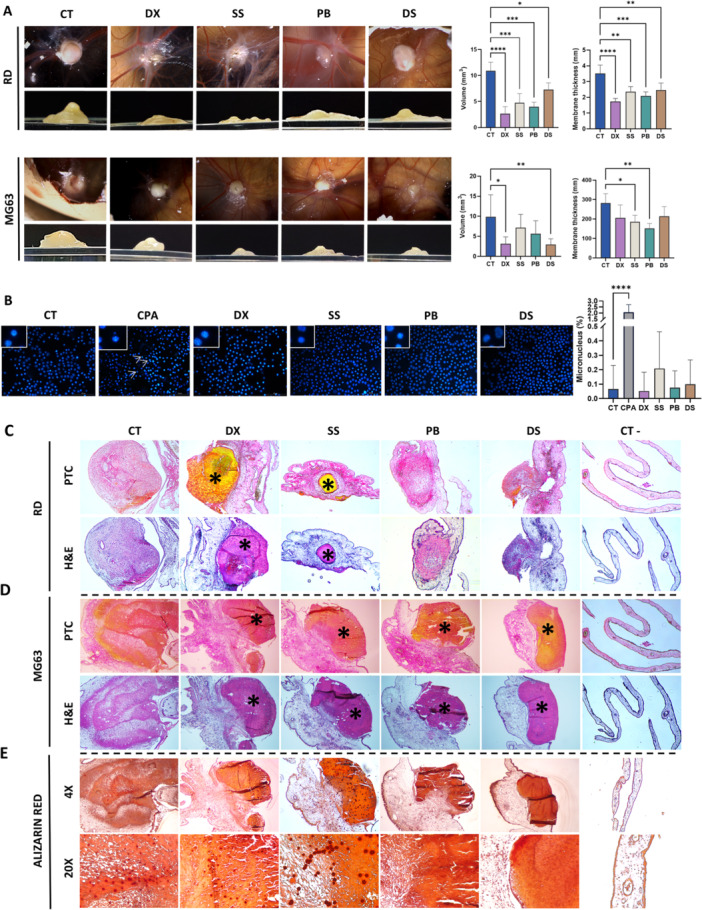
Effect of treatments on xenografts induced in the CAM using RD and MG63 cell lines. (A) Representative image of xenografts after 4 days of treatment, along with a graphical representation of tumor volume and membrane thickness. (B) Images of nuclei stained with Hoechst and a graphical representation of the percentage of micronuclei (40X). Arrows indicate the presence of micronuclei. Data are presented as mean values ± SD. Statistical significance relative to the control was determined as follows: *p < 0.05, **p < 0.01, ***p < 0.001, and ****p < 0.0001. Representative images (4x) of pentachrome staining (PTC) and H&E after 3‐day treatments with DX, SS, PB, DS or vehicle (CT) in CAM‐induced xenografts, and non‐tumor membranes (CT‐) in (C) RD and (D) MG63 cell lines are shown. (E) In MG63 tumors, alizarin red staining was also performed (4x and 20x). Asterisks (*) indicate necrotic areas composed of Matrigel and cellular debris.

In RD xenografts, all drugs significantly reduced tumor volume and CAM thickness, whereas in MG63, only DS and DX decreased volume (Figure 4A). None induced micronuclei formation compared to the CPA control (Figure 4B). Histology confirmed tumor development and treatment efficacy (Figure 4C,D); DX and SS induced extensive necrosis in RD, while SS promoted larger mineralized nodules in MG63 (Figure 4E), suggesting osteogenic differentiation. Conversely, PB and DS did not induce mineralization.

**FIGURE 5:**
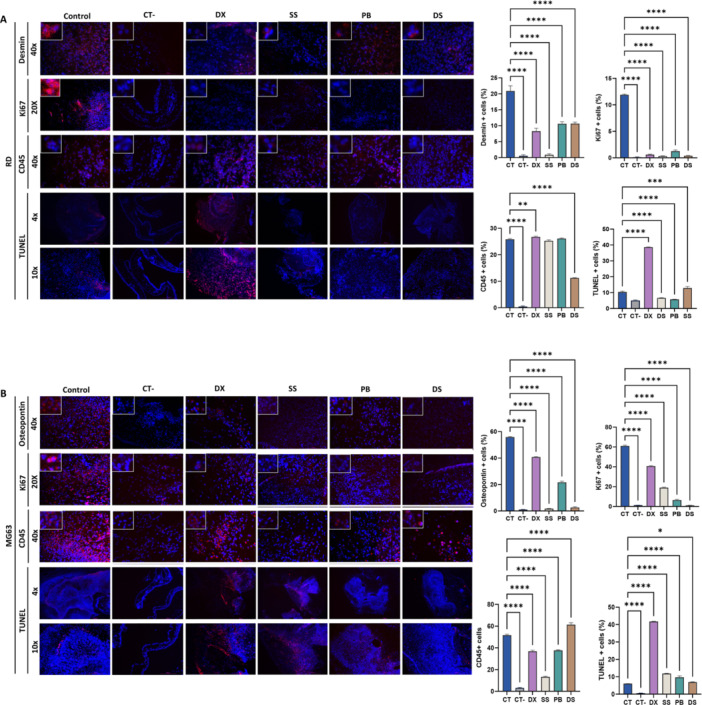
Modulation of marker expression in xenografts by treatments. Representative images from fluorescence microscopy analysis and graphical representation of Desmin (RD) and Osteopontin (MG63) markers, the cell proliferation marker Ki67, CD45, and the TUNEL assay in: (A) RD and (B) MG63 xenografts treated for 72 h with DX, SS, PB, and DS. Data are presented as mean ± standard deviation. Statistical significance was determined as follows: *p < 0.05, **p < 0.01, ***p < 0.001, and ****p < 0.0001.

Immunofluorescence (Figure 5A,B) showed decreased Ki67 and specific markers, desmin fell from 20.9% in RD, and osteopontin (55.8%) decreased in MG63 across all treatments. TUNEL assays confirmed DX‐induced apoptosis. Finally, DS reduced CD45+ cells in RD by 15.6%, while in MG63, SS and PB reduced them by 38.3% and 13.9%, respectively (Figure 5A,B).

## 4 Discussion

Osteosarcoma and rhabdomyosarcoma are aggressive pediatric cancers with high relapse rates, 30% and 40%, respectively (Defachelles et al. 2025; Fagioli et al. 2008). Given limited treatment efficacy, this study evaluates the antitumor potential of SS, PB, and DS as novel therapeutic and maintenance strategies for these sarcomas.

All three compounds showed in vitro antitumor activity. DS exhibited strong antiproliferative effects (IC_50_: 0.19–25.97 µM), consistent with Mandell et al. who reported an IC_50_ of 25 µM in Saos‐2 cells, which decreased to 1.2 µM upon the addition of copper (Mandell et al. 2022). SS showed IC_50_ values between 6.03 and 7 µM, in line with Yang et al. who reported a similar value (9.3 µM) in the synovial sarcoma cell line SW982 (Yang et al. 2018). PB demonstrated the lowest activity (13.25–69.77 µM), although previous studies suggest its potential in sarcomas with CDK4 overexpression (Iwata et al. 2021). These findings underscore the differential sensitivity of sarcoma cell lines to these three compounds.

Cell migration and colony formation are key markers of metastatic potential and mortality in sarcomas (Kelley et al. 2020). PB was the most effective treatment, reducing migration in all cell lines, consistent with other CDK4/6 inhibitors like lerociclib (Julson et al. 2024). Additionally, all compounds decreased colony formation, aligning with reports for PB in head and neck cancer lines (Li et al. 2024), while Liu et al. demonstrated that SS can reduce colony formation by 60% and approximately 50% in 789‐O and ACHN renal cell carcinoma cell lines (Liu et al. 2022). Likewise, Beaudry et al. reported that DS decreased colony formation by 55% in the N91 neuroblastoma cell line (Beaudry et al. 2023). Overall, SS, PB, and DS decreased cell aggressiveness, with PB being the most effective in all lines.

In 3D models, SS markedly reduced cell viability without altering spheroid volume, matching findings by Doello et al. (Doello et al. 2021). Mechanistically, cleaved PARP1 overexpression confirmed apoptosis in most cell lines (Bastos et al. 2024). Furthermore, improved viability following NAC co‐treatment and nuclear translocation of AIF demonstrated that SS and DS induce ROS‐mediated parthanatos, a caspase independent death pathway (Schiefer et al. 2024; Sun et al. 2025). These results confirm that while structural changes in spheroids may be absent, these compounds effectively trigger specific biochemical cell death pathways in sarcoma models.

Autophagy was identified as another cell death mechanism. Increased LC3β II expression and Lysotracker intensity in RD and Saos‐2 cells suggested autophagolysosome formation following treatment with DX, SS, and PB (Zhang et al. 2023). However, occasional discrepancies between these markers may reflect pH changes in acidic organelles detected by Lysotracker independently of autophagy (Barral et al. 2022). These findings indicate that while the drugs induce autophagy, the cellular response varies across conditions.

Interestingly, VEGFA is an angiogenic factor crucial for enhancing blood vessel formation and increasing vascular permeability, both necessary for tumor initiation and progression (Kang et al. 2024). In fact, high VEGFA expression has been correlated with poor prognosis and low survival rates in patients with solid tumors, including sarcomas (Lin et al. 2025). Similar to the antiangiogenic activity of SS in our study, other anti‐angiogenic drugs have demonstrated remarkable efficacy in sarcoma treatment. Apatinib, a VEGFR2 receptor inhibitor, is currently in a phase II clinical trial for untreated or chemotherapy‐refractory STS (Yu et al. 2022). Similarly, regorafenib, a multikinase inhibitor with anti‐angiogenic activity, has been approved for second and subsequent lines of treatment in advanced STS (Grothey et al. 2013).

CSC markers (ALDH, CD44, CD133) drive sarcoma progression and chemoresistance (Galoian et al. 2024; Chico et al. 2023). Our results suggest that SS may prevent the selection of CD44 overexpressing populations, which are linked to DX induced resistance and inhibited apoptosis (Gerardo‐Ramírez et al. 2022). Combining SS with other treatments could avoid selecting for these resistant markers. These differential sensitivities highlight the potential for personalized medicine through histological marker profiling or liquid biopsy to optimize sarcoma therapies.

Finally, drugs were evaluated using in ovo CAM tumor models, which provide a vascularized microenvironment ideal for sarcoma development (Ribatti and Annese 2023; Shoji et al. 2023). We successfully established RD and MG63 xenografts, where histological analysis revealed treatment‐induced necrotic areas. These findings, consistent with Shoji et al.'s results for vincristine, confirm the effectiveness of our treatments in a complex in vivo platform.

Given the osteoblast‐like properties of MG63 cells, these xenografts can form calcium‐rich nodules (Chatree et al. 2023). In this context, the CAM provides a complex microenvironment with blood supply, oxygen, electrolytes, and growth factors (Schneider‐Stock and Ribatti 2021). The observed mineralization suggests that certain treatments, such as SS, may promote osteogenic differentiation, in line with ongoing research on differentiation therapy for osteosarcoma. Osteoblast‐derived exosomes shown to inhibit proliferation and promote osteogenic differentiation (Leng et al. 2024). Additionally, chemotherapy response has also been correlated with tumor mineralization (Henderson et al. 2020). PB and DS did not induce mineralization, likely due to proliferation arrest prior to matrix formation.

Immunohistochemical analysis of RD‐derived xenografts revealed a marked reduction in Desmin, a rhabdomyosarcoma marker associated with poor prognosis (Godbole et al. 2006). Similarly, Osteopontin expression, a progression‐associated marker in osteosarcoma, was also decreased (Agrawal et al. 2024). In all cases, these changes were accompanied by a reduction in Ki67, a proliferation marker. Previous studies have linked Ki67 expression to adverse outcomes in sarcomas. Soffer et al. reported an association between Ki67 levels and lymph node dissemination in rhabdomyosarcoma (Soffer et al. 2019), while Zeng et al. found that high Ki67 in osteosarcoma correlated with distant metastasis and reduced overall survival (Zeng et al. 2021).

Inflammation in CAM xenografts prompted the evaluation of CD45+ infiltration. Although avian immune cells remain inactive until ED18 (Garcia et al. 2021), the increase in CD45+ cells in MG63 tumors is significant, as promoting immune infiltration can correlate with inhibited tumor growth (Wang et al. 2022). While these results are promising, the CAM model is a preliminary preclinical step; these findings require validation in complex mammalian models to better simulate the human tumor microenvironment.

## 5 Conclusions

This study demonstrated the antitumor activity of the FDA‐approved drugs PB and DS, as well as the non‐approved SS, in osteosarcoma and rhabdomyosarcoma cell lines in vitro. Additionally, these drugs inhibited cell migration and colony formation. They also induced different cell death mechanisms, including apoptosis and autophagy. Furthermore, SS and DS promoted ROS induction and parthanatos‐mediated cell death. In ovo studies further confirmed the antitumor potential of these drugs. In summary, SS, PB, and DS are promising therapeutic candidates for sarcoma treatment, supported by established safety profiles. Further studies are needed to determine their clinical application, even as maintenance therapies.

## Author Contributions

**María Ángeles Chico:** writing – original draft, validation, software, methodology, investigation, formal analysis. **Kevin Doello:** conceptualization, writing – original draft, validation, software, methodology, investigation, formal analysis. **Raul Ortiz:** methodology, investigation, supervision. **Mercedes Peña:** investigation, formal analysis, validation. **Consolación Melguizo:** conceptualization, writing – review and editing. **Cristina Mesas:** validation, investigation, formal analysis, writing – review and editing. **Jose Prados:** conceptualization, writing – review and editing. All authors read and approved the final manuscript.

## Funding

The authors have nothing to report.

## Conflicts of Interest

The authors declare no conflicts of interest.

## Supporting information

Supporting File 1

## Data Availability

The data that support the findings of this study are available from the corresponding author upon reasonable request. Data will be made available on request.
