# Supplementary material for: Antitumor Activity of Sodium Selenite, Palbociclib, and Disulfiram Against Osteosarcoma and Rhabdomyosarcoma Cell Lines
Source: Drug Dev Res. 2026 May 3;87:e70304. doi: 10.1002/ddr.70304 (PMC13135720; doi:10.1002/ddr.70304)
Supplement: Supplementary file 1 — Supporting File 1 [file DDR-87-e70304-s001.docx]

**Supplementary material**

**Antitumor activity of sodium selenite, palbociclib, and disulfiram against osteosarcoma and rhabdomyosarcoma cell lines**

**Antitumor Agents in Sarcoma Cell lines**

**María Ángeles Chico ^1, 2, 3^, Kevin Doello ^1, 2, 3, 4^, Raul Ortiz ^1, 2, 3^, Mercedes Peña ^1, 2, 3^, Consolación Melguizo ^1, 2, 3, *^, Cristina Mesas ^1, 2, 3, *^, Jose Prados ^1, 2, 3^**

^1^ Instituto de Investigación Biosanitaria de Granada, ibs.GRANADA, 18012 Granada, Spain

^2^ Institute of Biopathology and Regenerative Medicine (IBIMER), Center of Biomedical Research (CIBM), University of Granada, 18100 Granada, Spain

^3^ Department of Anatomy and Embryology, Faculty of Medicine, University of Granada, 18071 Granada, Spain

^4^ Medical Oncology Service, Virgen de las Nieves Hospital, 18016 Granada, Spain

**Table S1.** Primers used in the RT-qPCR analysis.

| **Gene** | **Gene Name** | **Primer sequence** |
| --- | --- | --- |
| GAPDH | Glyceraldehyde-3-phosphate dehydrogenase | F: TCGGAGTCAACGGATTTG  R: CAACAATATCCACTTTACCAGAG |
| ALDH1A1 | Aldehyde dehydrogenase 1 family member A1 | F: GACAATGGAGTCAATGAATGG  R: ATCAATTGGTATTGTACGGC |
| CD44 | CD44 molecule | F: TTATCAGGAGACCAAGACAC  R: ATCAGCCATTCTGGAATTTG |
| CD133 | Prominin 1 | F: CAGTATCAATTCAGTGCTAGG  R: CTTAATCTCATCAAGAACAGGG |
